# Cognitive Load Theory for debriefing simulations: implications for faculty development

**DOI:** 10.1186/s41077-018-0086-1

**Published:** 2018-12-29

**Authors:** Kristin L. Fraser, Michael J. Meguerdichian, Jolene T. Haws, Vincent J. Grant, Komal Bajaj, Adam Cheng

**Affiliations:** 1 Department of Medicine, Cumming School of Medicine, University of Calgary, 7007 14 St SW, Calgary, Alberta T2V 1P9 Canada; 2 Department of Emergency Medicine, NYC Health + Hospitals/Harlem Simulation Center, Columbia University School of Medicine, New York, USA; 3 Department of Medicine, Cumming School of Medicine, University of Calgary, Calgary, Canada; 4 Departments of Pediatrics and Emergency Medicine, Cumming School of Medicine, University of Calgary, Calgary, Canada; 5 New York City Health and Hospitals Simulation Center, New York, USA

## Abstract

The debriefing is an essential component of simulation-based training for healthcare professionals, but learning this complex skill can be challenging for simulation faculty. There are multiple competing priorities for a debriefer’s attention that can contribute to a high mental workload, which may adversely affect debriefer performance and consequently learner outcomes. In this paper, we conceptualize the debriefer as a learner of debriefing skills and we discuss Cognitive Load Theory to categorize the many potential mental loads that can affect the faculty debriefer as learner. We then discuss mitigation strategies that can be considered by faculty development programmes to enhance professional development of debriefing staff.

## Introduction

The adoption of simulation in healthcare has been fuelled by studies demonstrating enhanced participant knowledge, performance and, in some cases, a positive impact on patient outcomes [1, 2]. The debriefing that takes place following the clinical scenario has been shown to be a critical component in the learning process [3], but debriefing is a complex, dynamic skill that requires practice to achieve proficiency. Facilitators are challenged to perform multiple tasks in a short time frame such as observe participant behaviours, meaningfully structure the debriefing to encourage reflective discussion, and provide open, honest feedback while fostering a safe learning environment [4–9]. The complex cognitive processing places a significant demand on facilitators’ working memory [10] which is important because working memory has a finite capacity to process information. When the “bandwidth” available for processing novel information is exceeded, then performance can suffer and subsequently learning may be impacted. From the authors’ collective experience in faculty development for simulation debriefing both locally and internationally, we describe strategies that we have found helpful for teaching debriefers how to manage the high mental workload to improve the quality of their debriefings. To provide a theoretical basis for this discussion, we have framed these practices with the principles of Cognitive Load Theory (CLT).

CLT is an empirically derived theory that contends that learning can only occur if there is adequate room in working memory for processing of new information so that it can be stored in long-term memory. We argue that most facilitators are learners themselves [8, 11] and their ability to acquire the skills required for an effective debriefing will be impaired if the demands on working memory are too great. We believe that facilitators will be best positioned to develop and learn from their debriefing experiences if conditions for learning are appropriate (i.e. if there is adequate working memory bandwidth for that learning to occur). Although the importance of considering the cognitive load of learners in simulation has been described in the literature [12–15], the cognitive load of facilitators during debriefing has not been specifically discussed. In this paper, we apply the concepts of CLT to the complex task of debriefing and then propose some mitigation strategies that can be considered to reduce facilitator cognitive load and optimize opportunities for faculty development.

## Cognitive Load Theory

Cognitive Load Theory is a theory about how we learn, derived from our current understanding about limits of human cognition [16]. The framework has been applied to medical education in a variety of contexts and can be very useful for unpacking the complexity of learning tasks for the purpose of optimizing instructional design [17–20]. CLT contends that in order to learn something novel, the learner must attend to, manipulate and understand the information in a conceptualized “area” of the brain known as “working memory”. It is now well established that working memory is limited in both capacity and duration and if those limits are exceeded then learning is not guaranteed. While recognizing the limits of working memory, the CLT framework emphasizes that learning only occurs through the incorporation of new knowledge into complex schemata that can be accessed as single “chunks” of knowledge, in which case they will no longer impose any real load on working memory.

**Fig. 1:**
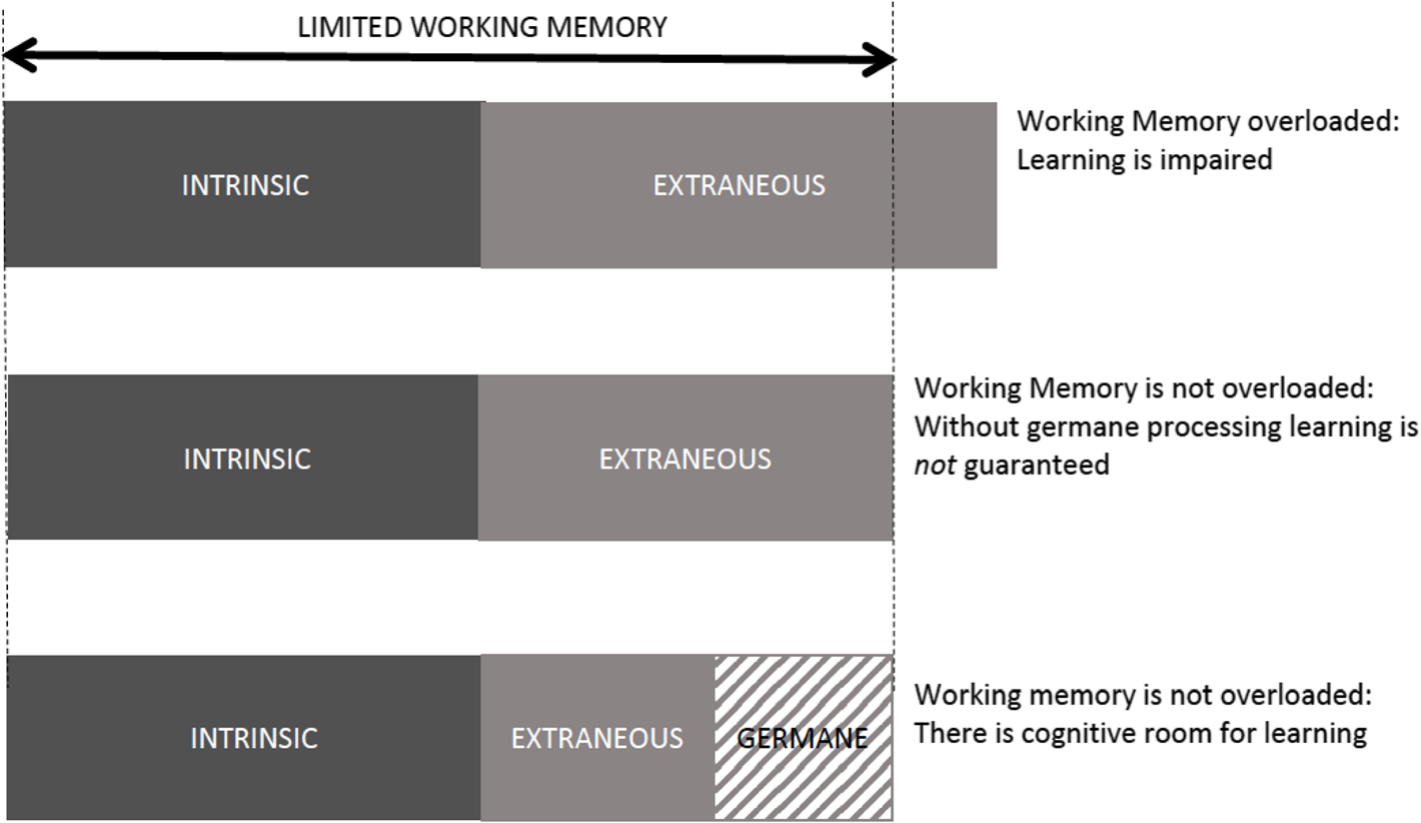
Effects of working memory limitations on learning

CLT describes three potential types of working memory loads, namely intrinsic, extraneous and germane [16] (see Fig. 1). *Intrinsic* load is due to the difficulty of the task, which is an interaction between expertise of the learner and complexity of information elements. When a learner comes across a new problem to be solved, the learner will often engage in inefficient problem solving techniques such as “means-end analysis” in which the learner thinks of all of the potential solutions to the problem and compares their possible utility to the problem at hand. But if a problem has been previously worked out, then the solution can be selected from long-term memory without much cognitive effort. Intrinsic load is therefore highly dependent on prior experience and the complexity of the problem (defined as the number of elements that need to be compared at once). *Extraneous* load is described as the mental processes that are imposed by poor instructional design and are not relevant for learning [21–24]. Some have described this as anything in the learning environment (i.e. noise) that distracts from the actual learning point. For example, several empiric studies have shown that when instructional materials are presented in a “split-attention” format (e.g. medical diagrams with explanatory text outside of the figure instead of embedded within) then the high cognitive demand of integrating the multiple sources of information can impair learning [18, 25]. The split attention effect is just one of many “effects” described by CLT that address the way in which information is presented to learners. In the context of simulation, it is helpful to consider the overall learning objectives of the scenario in deciding whether or not the presentation format is truly “extraneous”. For example, presenting information to a learner from more than one source (traditionally considered to be split-attention) might be desirable if having a learner integrate information from multiple sources (e.g. the parent, the patient and the electronic medical record) is relevant to clinical practice. However, for a junior learner where the objective is to diagnose pneumonia from the clinical signs and symptoms, searching in different places for this information would add cognitive effort that may not be directly relevant to the learning objective. It is important to recognize that the concept of extraneous load was originally described in relation to very simple learning environments such as solving math problems or learning about electrical circuits from a diagram [24]. The large number of empirical investigations supporting the “split attention effect” [26] suggests that this is likely relevant to other learning environments; however, its application to highly dynamic learning environments such as simulation or facilitation requires more study.

The concept of *germane* load was introduced to CLT in the mid-1990s when experimentalists found that introducing intentional variability to practice sessions actually improved learning despite an increase in overall cognitive load [12, 27]. Schnotz further conceptualized germane load as “those cognitive activities in working memory that aim at intentional learning, beyond that of simple task performance”[28]. Examples included a conscious search for patterns in learning materials, meta-cognitive processing to monitor learning, restructuring of a problem representation to improve the ability to solve a problem and mindful construction of cognitive schemata. Expertise is attained through the incorporation of new knowledge into these complex schemata.

More recently, some researchers propose that germane load is really a subgroup of intrinsic load since changing the nature of a task to encourage deeper processing (such as encouraging students to compare and contrast, or self-questioning techniques) is essentially changing the intrinsic difficulty of the task (i.e. the intrinsic load) [29]. This is an area of ongoing controversy and certainly this simplified classification of CLT may aid those who are trying to improve measurement and reconcile the paradoxical claim that some loads can actually be “good” for learning [30]. For the purposes of this paper, we find the notion of germane processing, that part of the task that encourages schema construction, to be a helpful category as we consider strategies that might improve learning by faculty debriefers.

## Cognitive loads of debriefing

Debriefing is a complex task and if the mental workload of the debriefing exceeds the cognitive capacity of the facilitator then performance can suffer. Becoming an expert facilitator requires time and practice; thus, it behooves us to ask: is there any cognitive room left for learning? When we consider the facilitator in simulation training as a “learner” themselves, excessive cognitive load during debriefing can impact their growth as emerging experts in debriefing. How can we support our debriefers in this complex environment so that they can still learn during debriefing activities? In the following section, we explore the potential loads that facilitators may have to manage during a debriefing.

Many tasks need to be carried out by the facilitator during a single simulation session, and these begin prior to the actual debriefing. During the pre-briefing, the facilitator needs to set the ground rules and objectives for simulation, take steps towards establishing a safe container for learning, establish the “fiction contract”, and identify potential threats to a successful debriefing [4]. Throughout the scenario, the student performance is being observed and compared to the expected or target behaviours for the learner group. Additional tasks are often assigned to facilitators, including managing technology (e.g. operating the mannequin, displaying audio-visuals) or playing an acting role within the scenario itself. Finally, the scenario concludes and the facilitator has a short time to formulate a plan for the debriefing. Below we will discuss some of the intrinsic, extraneous and germane loads that facilitators may encounter during the debriefing phase of simulation training (see Table 1). We have categorized these multiple working memory loads for the reader based on our experiences of “typical” scenarios; however, it is possible that a potential “load” that we have categorized under one category might also contribute load of another category. For example, co-debriefing is a strategy that is often used to reduce the cognitive load of a solitary debriefer by sharing the tasks of debriefing and especially providing back-up in the case of a particularly challenging clinical discussion or difficult learner dynamics. However, we have seen many situations in which a second debriefer can actually cause increased cognitive work to the primary debriefer if their respective roles have not been well defined or if they do not share a common mental model for the debriefing structure. Thus, this table is not meant to be exhaustive but provides common examples that we use in our faculty development programmes.

**Table 1:** Intrinsic, extraneous and germane loads during debriefing

	Drivers of load	Mitigation strategies
Intrinsic load: Related to task difficulty and debriefer expertise	Quantity of information: • Number of learners • Number of learning objectives • Various learner types/professions	Match debriefer expertise to size of groups and number of professions Use of whiteboard or flip chart for noting topics of discussion Share decision making with learners Co-debriefing to share cognitive load
	Emotional state of learners	Prebriefing to establish a “safe container” for learning
	Insufficient time for debriefing	Schedule adequate time Do not allow the scenario to run longer than scheduled
	Debriefer clinical expertise	Provide debriefers with detailed notes about clinical content Consider inviting a content expert (co-debriefing)
	Debriefer experience	Debriefing scripts for novice debriefers can help structure debriefing Debriefing assessment tools can function as “pre-learning” around expected facilitation skills
Extraneous load: Imposed by poor instructional design and not relevant for learning	Scenario realism needs to be addressed	Establish fiction contract prior to the simulation
	Debriefing going off topic	Debriefing framework can help organize debriefing for both the debriefer and for the learner Use previewing statements to introduce next topic of discussion
	Physical space: • Comfort • Ambient noise • Interruptions	Arrive early to set up debriefing space Seating arrangement for optimal eye contact Limit interruptions, coming and going of learners or observers
	Performance anxiety	Co-debriefing for back-up Establish a safe container for facilitators
	Difficult learner dynamic	Co-debriefer can monitor members of the group for distress /participation Facilitators can learn schemata for common learner dynamics (e.g. how to engage the quiet learner)
	Video review	Co-debriefer can be tasked to run video Strategic and selective use of video
	Co-debriefing • Interruptions • Competing priorities	Facilitator pre-brief to set expectations/roles/goals Co-debriefers utilize strategies such as open negotiation and permissible interruptions
	Evaluation of learners	Only add concomitant learner evaluation for very experienced facilitators
Germane load: Conscious, effortful attempts to learn from the debriefing experience	Reflection in action: • Are all learners engaged? • Are all of the objectives covered?	Do not be afraid to pause to make a mental or physical note of a successful strategy used or pitfall to avoid
	Reflection on action: • Review of learner feedback • Make notes for future debriefing structure, content or approach	Peer to peer coaching in a safe learning environment Obtain debriefing feedback from learners Make use of published tools for assessing debriefing Provide protected time for reflection to occur

## Intrinsic load of debriefing

Once learners are finished with the scenario, the facilitator is typically expected to carry out the following tasks, all of which are components of the intrinsic load: (1) recall what happened in the scenario and review the case with the learners; (2) prioritize topics for discussion; (3) formulate questions that will encourage active participation of all learners; (4) listen to learner responses to gage and manage emotions (5) categorize responses to develop an efficient learning plan to address essential objectives and any emerging learner concerns; (6) ensure that teaching is effective and clear, and that performance gaps are closed; (7) manage learner emotions and cognitive load (8) ensure learner psychological safety, (9) manage own emotions and (10) manage own preconceived notions (i.e. frames) about the learners and their actions. The degree of intrinsic load imparted by these tasks will vary depending on several factors, including the expertise and knowledge of the facilitator, the degree of engagement of the learners, and the nature of the performance.

## Extraneous loads of debriefing

Extraneous loads are those working memory resources required for task completion that do not enhance learning. Because running a simulation and the associated debriefing can be highly dynamic and unpredictable, it can be especially challenging to proactively manage all potential extraneous loads:

*Facilitator role*: While it is tempting for facilitators to perform “just a few” extra tasks in addition to debriefing (e.g. navigating video replay, keeping track of time), one needs to remember that these activities require mental effort that is extraneous to learning the skills of debriefing.

*Co-facilitation*: Co-debriefing with a colleague can be a useful strategy to reduce the intrinsic load on the primary facilitator by sharing tasks, but it can paradoxically add significant extraneous load [31]. If the second facilitator holds a different mental model for how the debriefing will run, then the primary facilitator ends up with the added mental task of redirecting the discussion, or potentially managing interruptions triggered by the secondary facilitators. Additionally, negotiating the way forward with their co-debriefer in a professional, respectful way without squandering valuable time can be challenging. These additional loads on working memory are particularly prominent when co-debriefers have not had a chance to discuss their facilitation strategy prior to the debriefing.

*Learners:* Another source of extraneous load can be from learners, who may focus on other aspects of the simulation apart from the intended debriefing goals. For example, complaints about the simulation scenario design or realism are not uncommon. Addressing these learner concerns is paramount to maintaining rapport and trust [32]; however, it can also consume valuable facilitator mental resources especially when a facilitator might feel somewhat defensive about the scenario that they just facilitated. Other learner behaviours that can add workload to the debriefing include conversations that are tangential to the intended purpose of the discussion, or overly emotional learners. Facilitators can then have their own emotional reactions (including performance anxiety) related to managing learner reactions that will add to the cognitive demands of debriefing.

## Germane load during debriefing

During any debriefing, the facilitator will experience some things that go well and some that do not go as well. For example, a novice facilitator might notice that they have difficulty keeping the discussion on track. Recalling, in the moment, that the preceding reaction phase did not include all participants, the facilitator can consider that this might be contributing to the students’ ongoing interruptions of each other. The facilitator realizes that ensuring a complete reaction phase in which all learners have a chance to “vent” their reactions can reduce the likelihood of such tangential discussions in future. Thus, they learn from experience during the debriefing through in-action reflection [33, 34], and the mental effort required to do so is classified as germane load. Similarly, a facilitator might be having difficulty getting students to reveal their perspectives on a case so they try a new way of phrasing a question and notice that this yields fruitful discussion; then, they make a mental note to add it to their repertoire for future discussions. Such reflection-in-action is a powerful way for improving performance in the moment, but also for future performance and growth.

Alternatively, the facilitator who recognizes a debriefing challenge might make a mental note to seek feedback from a peer or an expert after the session [35]. The cognitive effort required to commit to finding solutions later is also a type of germane processing, but in this case, the majority of the mental effort can be delayed to a later time when there will likely be more cognitive room available for meaningful learning (i.e. reflection-on-action). To optimize these opportunities for improving their debriefing skills, facilitators should look to employ strategies to mitigate the intrinsic and extraneous loads during debriefing.

## Mitigation of cognitive load during debriefing

### Managing intrinsic load

One proactive strategy to reduce intrinsic load during debriefing is to match the debriefing difficulty to the skill level of the facilitator. Ideally, a programme can anticipate challenging debriefings and allocate them to more experienced facilitators [36]. The facilitator can also proactively minimize debriefing difficulty by ensuring that the number and content of objectives for the scenario are well aligned with learner experience and profession [22]. Task complexity of the debriefing also increases when the scope of the clinical content being covered is outside the expertise of the facilitator. Ideally, clinical content should be reviewed in detail prior to the session to ensure that the facilitator has easy access to clinical schemata in long term memory [21, 37]. Alternatively, the potential intrinsic load of difficult medical content can be reduced by inviting a subject matter expert to attend the debriefing, or by providing supplemental handouts to learners.

Sharing clinical learning and decision-making with the learners can potentially reduce intrinsic load. Often times, there are learners with clinical expertise that is equal to or greater than that of the debriefer. Encouraging learners to share their knowledge about the subject can reduce workload on the debriefer while encouraging a more learner-centered type of discussion. Learner-centered debriefing strategies, like the plus-delta method, allow learners to choose topics that they find most relevant for reflection [38, 39] and can thereby reduce the burden on the facilitator to decide the course of the conversation [40]. Of course, allowing learners too much leeway may lead to off-topic discussions which can further challenge the facilitator; so the implementation of strategies to mitigate load also require practice.

One strategy that is often used to support new faculty in their debriefing task is the provision of a framework to guide the debriefing [6, 7, 9, 41, 42]. Choosing a single strategy will reduce the mental workload of having to decide on the “flow” of conversation during the debriefing. Such frameworks have been adapted into cognitive aids for use by novice debriefers during debriefing [43, 44]. After practice with a particular framework, the approach can be automated and stored in long-term memory (as a single schemata) for the debriefing conversation [43]. Training of faculty on the use of such aids to the point of some automation is essential so that the burden of the aid does not actually add to mental workload [45, 46].

### Managing extraneous load

Strategies to anticipate and reduce extraneous cognitive load should also be proactively considered. Dealing with defensive or argumentative learners can pose a significant and often preventable workload on the facilitator. Prior to the debrief, the facilitator should take steps towards creating a “safe container” for learning, where expectations and logistics are communicated, the fiction contract is explained and psychological safety is established [4]. If learners are not feeling threatened by the discussion about their good or poor performance, they will be more engaged in learning from experience rather than defending their practice. Distractions such as phone calls and participants/observers entering or exiting during the debriefing are other more obvious, preventable extraneous loads that should be proactively addressed.

Facilitator emotions can impose extraneous workload that may affect cognitive processing and hinder effective learning from the debriefing [47, 48]. Feeling overwhelmed by the many debriefing tasks or having a weak understanding of the content of the case are examples of situations that can create anxiety. By sharing the debriefing with a co-facilitator, the risk of being overwhelmed is potentially diminished by having “back-up” support. Co-debriefing offers the benefits of pooled expertise, shared monitoring of many learners in the room, and a “lifeline” in the event of difficulties [31]. However, co-debriefing may also itself pose an extraneous load as discussed above. Strategies for effective co-debriefing that may reduce extraneous workload are conducting a facilitator pre-briefing, using non-verbal communication, previewing, and huddling with your co-debriefer to review facilitation performance after the debriefing is over [31].

### Optimizing germane load

The CLT framework emphasizes that learning really only occurs when essential schemata are constructed or elaborated in long-term memory; there must be adequate cognitive room for germane processing or learning will not occur. If we successfully mitigate debriefer mental workload as discussed above, we can then consider strategies to encourage germane processing about debriefing. Debriefers must be motivated to engage in germane processing [49] and accordingly, faculty development requires a supportive culture in which it is clear that self-reflection, peer coaching and expert feedback are valued. The process of self-reflection can be promoted by encouraging debriefers to review debriefing assessment tools (e.g. Debriefing Assessment for Simulation in Healthcare [DASH] or Objective Structured Assessment of Debriefing [OSAD]) and/or debriefing checklists prior to or after the debriefing [50–52]. Peer coaching and/or expert debriefing of the debriefer can also be framed by these assessment tools (e.g. DASH Rater Version)[53] to highlight desirable debriefing behaviours and to support the development of schemata to improve future performance.

## Limitations and future directions

While there is substantial evidence supporting the application of CLT to many disciplines, there is little data regarding best practices to manage cognitive load of faculty debriefers. In our experiences teaching debriefing courses, we have encountered many educators who feel that debriefing is overwhelming and difficult to learn in spite of our best efforts at faculty development. We believe that specific discussion of the various potential loads on the facilitator and associated mitigation strategies helps our faculty to manage the debriefing to optimize educational outcomes for learners and hopefully also for themselves. The suggestions that are made here are based on our collective experience, and we acknowledge the lack of empirical evidence to support these claims. While this is a limitation of this paper, the proposed strategies are intuitive, easy to implement, and highly unlikely to cause any harm. Future studies framed by a CLT theory would be helpful to determine which strategies are actually most important for improving faculty learning about debriefing and moreover educational outcomes for the learners of those faculty (Table 2).

**Table 2:** Cognitive load and debriefing—research opportunities

How do intrinsic, extraneous and germane load of debriefers vary during the phases of debriefing? How does this change with debriefing practice?	
What activities during the debriefing increase the cognitive load of debriefers?	
What strategies are most effective at reducing the intrinsic and extraneous loads of debriefers?	
Does the use of debriefing assessment tools (before or after debriefing) increase extraneous load or optimize germane load?	

We encourage simulation programmes to discuss CLT with their facilitators because this is increasingly recognized to have important implications for scenario design and learner outcomes. However, facilitators should also be encouraged to consider their own mental workload during debriefing, and how this might impact their ability to climb the learning curve of debriefing skills. They should strategize to mitigate their own cognitive load to optimize conditions for learning this challenging skill, to the ultimate benefit of their own simulation learners.

## Abbreviation

CLT: Cognitive load theory
